# Introductory Address

**Published:** 1842-09

**Authors:** 


*Introductory Address.*—Our readers will peruse with pleasure the address of Dr. E. Parmly, published in the present number of the Journal. The entertaining and instructive reminiscences which he relates are so intimately associated with recollections of our own, that they fall upon our ear as old familiars, and we doubt not that many who have struggled up the rugged path that leads to professional success, have encountered adventures differing from those described in importance but little. We would remark, however, that the ambition that prompted the writer to travel in pursuit of knowledge, and to toil unceasingly for its attainment, that he might be enabled to compete with the most excellent specimens of art that he had seen—that ambition should be emulated by every young practitioner of dental surgery. The nature of our profession is such that inferiority cannot but be regarded as disgrace. There is a point the attainment to which may be designated the mastery of the art. Every dentist should aim at this, and to those who lack the industry or ability to reach it, or in other words, who will not determine on doing so, we would say, and with feelings of the greatest kindness, desist wholly from a pursuit that will bring you nought but chagrin and disappointment. No medium course will do.

The remarks made by our author on the degree of mechanical skill requisite for the successful prosecution of the dental profession are correct and judicious; and he also very properly alludes to the inadequacy of mere mechanical skill. Who is there amongst us that has not seen the worst of conquences resulting from attempts at surgical operations made by self-entitled dentists, whose sole claims to the name were based upon a slight amount of skill picked up in the mechanical work-room of some scientific practitioner? And yet how many regard this kind of skill as ample proof of competency! Few practitioners can spare the time requisite to teach every branch of the art; and it is therefore gratifying to know that the attention of the profession is being directed to the importance of furnishing the necessary facilities for the acquirement of a complete and thorough knowledge of them.

